# Transcriptome profile analysis in spinal cord injury rats with transplantation of menstrual blood-derived stem cells

**DOI:** 10.3389/fnmol.2024.1335404

**Published:** 2024-02-01

**Authors:** Longju Qi, Wenwei Jiang, Wenhua He, Xiangzhe Li, Jiahuan Wu, Shiyuan Chen, Zehua Liao, Shumin Yu, Jinyi Liu, Yuyu Sun, Qinfeng Wu, Chuanming Dong, Qinghua Wang

**Affiliations:** ^1^Laboratory Animal Center, School of Medicine, Nantong University, Nantong, Jiangsu, China; ^2^Affiliated Nantong Hospital 3 of Nantong University, Nantong, Jiangsu, China; ^3^Rehabilitation Medicine Center, Suzhou Hospital, Affiliated Hospital of Medical School, Nanjing University, Suzhou, Jiangsu, China; ^4^Department of Basic Medicine, Luohe Medical College, Luohe, Henan, China

**Keywords:** spinal cord injury, non-coding RNA, competing endogenous RNA, axonal regeneration, menstrual blood-derived stem cells

## Abstract

**Introduction:**

Menstrual blood-derived stem cells (MenSCs) are vital in treating many degenerative and traumatic disorders. However, the underlying molecular mechanisms remain obscure in MenSCs-treating spinal cord injury (SCI) rats.

**Methods:**

MenSCs were adopted into the injured sites of rat spinal cords at day 7 post surgery and the tissues were harvested for total RNA sequencing analysis at day 21 after surgery to investigate the expression patterns of RNAs. The differentially expressed genes (DEGs) were analyzed with volcano and heatmap plot. DEGs were sequentially analyzed by weighted gene co-expression network, functional enrichment, and competitive endogenous RNAs (ceRNA) network analysis. Next, expression of selected miRNAs, lncRNAs, circRNAs and mRNAs were validated by quantitative real-time polymerase chain reaction (qRT-PCR). Bioinformatics packages and extra databases were enrolled to scoop the genes functions and their interaction relationships.

**Results:**

A total of 89 lncRNAs, 65 circRNAs, 120 miRNAs and 422 mRNAs were significantly upregulated and 65 lncRNAs, 72 circRNAs, 74 miRNAs, and 190 mRNAs were significantly downregulated in the MenSCs treated rats compared to SCI ones. Current investigation revealed that MenSCs treatment improve the recovery of the injured rats and the most significantly involved pathways in SCI regeneration were cell adhesion molecules, nature killer cell mediated cytotoxicity, primary immunodeficiency, chemokine signaling pathway, T cell receptor signaling pathway and B cell receptor signaling pathway. Moreover, the lncRNA-miRNA-mRNA and circRNA-miRNA-mRNA ceRNA network of SCI was constructed. Finally, the protein-protein interaction (PPI) network was constructed using the top 100 DE mRNAs. The constructed PPI network included 47 nodes and 70 edges.

**Discussion:**

In summary, the above results revealed the expression profile and potential functions of differentially expressed (DE) RNAs in the injured spinal cords of rats in the MenSCs-treated and SCI groups, and this study may provide new clues to understand the mechanisms of MenSCs in treating SCI.

## Introduction

Spinal cord injury (SCI) is a complex central nervous system disease that has emerged as a major global health issue. The global incidence of SCI is estimated to be around 500,000 cases annually, and this number continues to rise (Karsy and Hawryluk, 2019). The primary function of the spinal cord is to transmit sensory and motor signals between the brain and other parts of the body (Akinrodoye and Lui, 2021). As such, SCI is one of the most severe types of injury that can occur to the central nervous system. Various factors, including trauma, falls, collisions, spinal cord infections, or tumors, can cause spinal cord injuries, and it results in motor, sensory, and sphincter disorders below the level of the injury (Patil et al., 2015; Hamid et al., 2018). SCI can also lead to pressure ulcers, neuropathic pain, and a range of complications related to urinary dysfunction. Due to the loss of nerve tissue, the recovery of nerve function is limited, which negatively affects synaptic remodeling and neural circuit regeneration. Following an SCI, cavities or scars are formed by an influx of inflammatory cells at the injury site, which can impair the healing of the SCI. Despite intense research efforts and significant interest in the treatment of SCI, there have been relatively few significant breakthroughs that have translated into clinical applications (Freund et al., 2019). As such, the prevention, treatment, and cure of SCI have become a key concern for contemporary society, given the burden it places on families and communities.

Spinal cord injuries are typically classified into two stages: acute and chronic (Anjum et al., 2020). Cell-based therapy, particularly in the subacute stage, has emerged as an effective treatment for promoting neuronal recovery and nerve regeneration (Nishimura et al., 2013). Menstrual blood-derived stem cells (MenSCs) are a new type of mesenchymal stem cells with promising therapeutic effects in animal models and clinical trials (Chen et al., 2019; Zhu et al., 2019). In a previous study, we found that MenSCs transplantation in rat SCI models resulted in significant functional recovery and reduced inflammation and lesion cavities (Wu et al., 2018). However, the underlying mechanisms of MenSC therapy remain unknown. Recent developments in bioinformatics have shed new light on protein-coding mRNAs and non-coding RNAs (ncRNAs) and their role in SCI (Li et al., 2019; Peng et al., 2020; Salah et al., 2020; Wang J. et al., 2020). Molecular changes, including microRNAs (miRNAs), long ncRNAs (lncRNAs), and circular RNAs (circRNA), have been reported to play important roles in the pathophysiology of SCI, affecting various physiological processes such as cell proliferation, differentiation, apoptosis, and synaptic plasticity (Li M. et al., 2018; Zhang S. B. et al., 2019). Understanding these molecular changes may help in elucidating the therapeutic mechanisms of MenSCs and inform the development of more effective treatments for SCI (Chen and Schuman, 2016).

Non-coding RNAs (ncRNAs) are a crucial type of RNA that are involved in various genetic and epigenetic processes such as transcriptional and post-transcriptional regulation (Geng et al., 2018; Panni et al., 2020). Interestingly, despite the fact that only 10% of the genome codes for proteins, most of it is transcribed into functional non-coding RNAs (Zhao et al., 2020). NcRNAs can be classified into basic structure and regulatory types, and they play a significant role in both physiological and pathological processes (Li M. et al., 2018), including the regulation of neuropathic pain and activation of Wnt/β-catenin signaling in the spinal cord (Sun et al., 2018; Cui et al., 2019; Zhang S. B. et al., 2019). Due to their potential as diagnostic biomarkers and therapeutic targets for various diseases, ncRNAs are currently a popular research area.

In our study, we utilized RNA-seq technology to analyze the differentially expressed genes (DEGs) in the epicenter of the spinal cord in a hemisection rat SCI model, and predicted their functions in order to better understand the potential diagnostic, prognostic, and therapeutic value of these DEGs. Notably, lncRNAs and circRNAs can contain common miRNA response elements (MREs), and thus act as sponges that competitively bind with miRNAs through these MREs, thereby regulating the expression of target gene transcripts and affecting each other. These RNAs can form competing endogenous RNA (ceRNA) networks in human diseases, mediated by miRNAs. Given the potential roles of ceRNA networks in SCI progression, it is important to comprehensively elucidate the interactions between lncRNA/circRNA and miRNA, which may lead to promising biomarkers for the diagnosis and treatment of SCI (Hansen et al., 2013).

In this study, we conducted a comprehensive analysis of the transcriptome of the injured spinal cord epicenter in MenSCs-treated SCI rat models and control groups. We examined the expression profiles of various genes, including lncRNAs, circRNAs, miRNAs, and mRNAs, and identified DEGs through heatmap and volcano plot analyses. Additionally, the biological functions of DEGs by GO and KEGG pathway analyses. We also established PPI and ceRNA networks to investigate potential mechanisms underlying the therapeutic effects of MenSCs in SCI. These findings provide valuable insights into the pathophysiology of SCI and suggest potential novel targets for SCI treatment.

## Materials and methods

### Animals and MenSCs

In this work, 24 mature female wildtype Sprague Dawley rats (8Wks, 220–250 g) were purchased from the Experimental Animal Center at Nantong University [Breeding certification: SCXK(Su)2019-0001], rats were randomly assigned to the SCI and MenSCs therapy groups, and were raised in a pathogen-free environment with optimal temperature and humidity [Use license: SYXK(Su)2017-0046]. The rats had a normal immunological status and had not been treated earlier. All experimental techniques were overseen by the Institutional Animal Care and Use Committee of Nantong University (Approval No.: IACUC20190709-011). The animals were kept on a light/dark cycle of 12 h with free access to food and water.

As described before (Wu et al., 2018), human menstrual blood-derived mesenchymal stem cells (MenSCs) were obtained from adult women (between the ages of 28 and 38) after obtaining informed consent and following established ethical guidelines. The sample collection, cell culture, and transplantation procedures were approved by the ethics committee of Nantong Maternal and Child Health Hospital (Approval No.: 2016-023). The MenSCs used for transplantation were in their sixth to eighth generation.

### Surgery procedure of spinal cord injury

**FIGURE 1:**
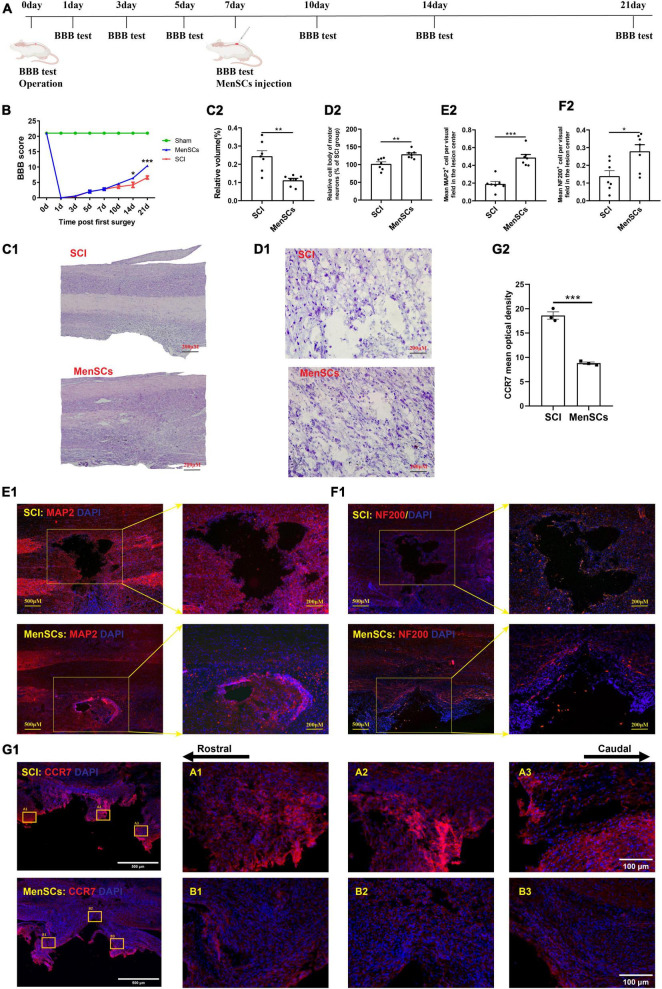
MenSCs treatment reduced the area of spinal cord lesions, and improved the recovery of motor function. **(A)** Flow chart of the experiment. **(B)** BBB scores indicate the motor functional index after SCI and MenSCs treatment. **(C1)** H&E staining of spinal cord samples from the SCI group and MenSCs-treated group at day 21 post surgery. **(C2)** Relative volume of injured area in panel **(C1)**. **(D1)** Nissl staining of spinal tissues containing lesion sites in SCI and MenSCs-treated rats. **(D2)** Relative cell body and morphology of motor neurons in the lesion sites in panel **(D1)**. **(E1)** MAP2 staining in SCI and MenSCs-treated rats’ spinal cords with lesion core. **(E2)** Mean MAP2^+^ cell per visual field in the lesion center in panel **(E1)**. **(F1)** NF-200 staining in SCI and MenSCs-treated rats’ spinal tissues with lesion core. **(F2)** Mean NF-200^+^ cell per visual field in the lesion center in panel **(F1)**. *n* = 7. **(G1)** CCR7 staining in SCI and MenSCs-treated rats’ spinal cords at the lesion site. **(G2)** Mean optical density of CCR7 per visual field in the lesion center in panel **(G1)**. *n* = 5. *P < 0.05, **P < 0.01, ***P < 0.001.

As indicated in our prior work (Wu et al., 2018), a thoracic 10 (T10) hemisection SCI was performed. An expert in the SCI rat model performed the hemisection surgery to eliminate the mistake induced by the incision. Briefly, rats were sedated by inhaling 2% isoflurane in oxygen-enriched air, and their eyes were lubricated with eye ointment. The rats were then injected subcutaneously with 2 mL of saline to restore the body’s fluid equilibrium. At T10, a laminectomy was performed to expose the spinal cord. Through a longitudinal incision in the dura, 0.5 cm of spinal cord was exposed. The spinal cord was severed with scalpel number 11 (Jinzhong, Shanghai, China). The incision was perpendicular to the central vessel and not transverse to the spinal cord’s central axis. In the sham group, just laminectomy on the T10 lamina was performed, with no following incision damage. The skin was stitched shut with 3-0 thread. The rats were then kept overnight on the warm pad, with only one-third of the cage on the pad for recuperation. For pain relief, slow-release buprenex was administered subcutaneously at a dosage of 0.05 mg/kg and penicillin was administered intramuscularly (i.m.) at a dose of 20 IU/day for 7 days after surgery. Twice a day, the rats’ bladders were compressed to facilitate urination until they recovered. Rats were observed every day to prevent weight loss, abnormal wound healing, and infection. In order to avoid the peak of immune response and enhance the residence and viability of MenSCs, the MenSCs treatment group was injected with 2.5 × 10^5^ cells using a microinjector at the rostral and caudal 2 mm of the injury site at day 7 post SCI (Figure 1A; Hwang et al., 2014). As described above, animals were nurtured and cared for carefully. Two weeks following the second operation, the rats were euthanized and the 5 mm long epicenter of the T10 spinal cord was taken for sequencing.

### Pathological investigation

The spinal cord samples were embedded in OCT and longitudinally sliced into 20 μm sections for hematoxylin and eosin (H&E) staining. Specifically, a series of sections were stained with hematoxylin for 2 min and 40 s, washed twice with distilled water, differentiated with 1% hydrochloric acid for 30 s, washed twice with distilled water, and stained with eosin for 1 min. The slides were then covered and images were captured for subsequent analysis.

Nissl staining was carried out with Nissl stain kit (G1430) produced by Solarbio Life Sciences. Under the instruction of the manufacture, cresyl violet method was adopt to reveal the morphology of neurons. Images were acquired by microscope (Olympus DP71, Tokyo, Japan) and processed with Image J software.

For immunofluorescence, sections were soaked with PBS solution containing 3% normal donkey serum (NDS, Jackson) and 0.3% Triton X-100 (Sigma-Aldrich, St. Louis) to block the extra antigens at room temperature for half an hour. And then incubated with specific primary antibodies MAP2 (Millipore; 1:1000), NF200 (Abcam; 1:1000), and CCR7 (SAB, 1:200) at 4°C overnight. Sections were consequently washed with PBS for 10 min × 3 times, and then labeled with secondary antibodies such as Goat anti-mouse, Alexa Fluor^®^ 488 (Invitrogen; 1:1000) and Goat anti-rabbit, Alexa Fluor^®^ 488 (Invitrogen; 1:1000) for 1 h at room temperature. Hoechst 33342 (Sigma, 1:1000) was used to reveal cell nuclei. Images were harvested by fluorescence microscope (Olympus BX 51, Tokyo, Japan). Pictures were analyzed with Image-Pro Plus 6.0.

### Behavioral assessment of locomotor recovery

Locomotor recovery was assessed using the Basso, Beattie, and Bresnahan (BBB) locomotor rating scale, which has been previously described. Two well-trained experimenters who were blinded to the treatment groups conducted independent scoring according to the BBB scale (Basso et al., 1995).

### RNA extraction and sequencing

In all 3 experimental groups, a 5 mm spinal cord sample was collected from the site of injury, flash-frozen, and stored in liquid nitrogen. Total RNA was extracted using the TRIzol reagent kit (Invitrogen, Carlsbad, CA, USA), and small RNA molecules within the 18–30 nt range were enriched using polyacrylamide gel electrophoresis (PAGE). cDNA, DNA, and small RNA libraries were prepared and sequenced on the Illumina sequencing platform by Genedenovo Biotechnology Co., Ltd. (Guangzhou, China). Short reads alignment tool Bowtie2 (version 2.2.8) was used for mapping reads to ribosome RNA (rRNA) database. An index of the reference genome was built, and paired-end clean reads were mapped to the reference genome using HISAT2 (Patil et al., 2015) (version 2.1.0) with “-rna-strandness RF” and other parameters set as a default. The reconstruction of transcripts was carried out with software Stringtie (version 1.3.4), all of the reconstructed transcripts were aligned to reference genome and were divided into twelve categories by using Cuffcompare. And the Ensembl_release98 was used as reference genome. The total read values were 66850386, 62865432, and 67397652 in control group, 59868336, 77135594, and 84628202 in SCI group, 73572850, 72903728, and 89670958 in MenSCs group; the mapped read values were 64381941, 60586983, and 64884601 in Control group, 57555397, 74520157, and 81795491 in SCI group, 71152084, 70299993, and 86499162 in MenSCs group. The edgeR software package^1^ was employed to identify differentially expressed transcripts (DEGs) between different groups. DEGs were defined as mRNAs and ncRNAs with a fold change ≥ 2 and a false discovery rate (FDR) < 0.05. Additionally, three software programs (mireap, miRanda, and TargetScan) were used to predict miRNA targets, with miRNA sequence and family information obtained from the TargetScan website.^2^

### qRT-PCR validation

Total RNA was extracted from spinal cord samples using TRIzol reagent (Pufei Biological, USA) following the manufacturer’s instructions. The RNA was then reverse transcribed into cDNA using a reverse transcription kit (Vazyme, Nanjing, China). The expression data was normalized to the expression of Gapdh using the 2^–ΔΔCt^ method.

### GO and KEGG enrichment analysis

Gene ontology analysis for DE mRNAs were performed to construct gene annotations from the perspective of biological process (BP), cellular component (CC) and molecular function (MF). All DEGs were mapped to GO terms in the Gene Ontology database,^3^ gene numbers were calculated for every term, significantly enriched GO terms in DEGs comparing to the genome background were defined by hypergeometric test. The *P-*value in GO analysis is used to test the reliability of the analysis, and the top 20 terms in each category (according to the *P-*value) are displayed as a bar graph. KEGG analysis performed with Omicshare platform of Genedenovo helped us to further understand genes biological functions and interactions among differentially expressed genes and the top 20 pathways were presented in bar graph. All DEGs were mapped to the KEGG^4^ pathways.

### Establishment of the ncRNA–miRNA–mRNA network

The competing endogenous RNA (ceRNA) network was generated by identifying potential target mRNAs and non-coding RNAs (ncRNAs) for the miRNAs of interest. The interactions between these RNA molecules were visualized using Cytoscape 3.8.1 software.

### Construction of PPI network and identification of hub genes

Construct the PPI network of the DE mRNAs encoding protein through the online database STRING^5^ and visualize it with Cytoscape 3.8.1 software (Doncheva et al., 2019). Subsequently, cytoHubba app of Cytoscape was used to determine hub genes. The top 10 nodes ranked by MCC topological analysis, and display options set as check the first-stage nodes, display the shortest path, and display the expanded subnetwork. And use the software as follow, click Nodes’ Scores/Calculate/Select nodes/Hubba notes/Top 50 node(s) ranked by MCC.

### Statistical analysis

The GraphPad Prism software was utilized for statistical analysis. All information was presented as the mean standard deviation (mean ± SD). The Student’s *t*-test was performed to evaluate statistical differences between the two groups, and *P* < 0.05 was considered statistically significant.

## Results

### The influences of MenSCs on treatment of SCI

In this study, the Basso-Beattie-Bresnahan (BBB) score indicated an improvement in motor function recovery following MenSCs treatment (Figure 1B). Furthermore, we employed hematoxylin and eosin (H&E) staining at 21 days post-surgery to evaluate the efficacy of menstrual blood-derived stem cell (MenSCs) treatment on spinal cord lesions, which revealed a reduction in lesion volume (Figures 1C1, C2). The results of the Nissl staining showed that MenSCs treatment positively increased the cell body area and morphologic characteristics of motor neurons in MenSCs-treated rats than that in SCI rats in the injured sites (Figures 1D1, D2). Next, to determine the effect of MenSCs treatment on neuronal viability after injury, we performed MAP2 staining and results illustrated that there were more MAP2-positive mature neurons in the lesion area at MenSCs adoption rats in comparison with the SCI ones (Figures 1E1, E2). Immunostaining results exhibited that there were higher numbers of NF-200-labeled axonal fibers in the lesion sites in the MenSCs group than the SCI group (Figures 1F1, F2). Significantly lower expression of CCR7 was observed in the MenSCs group than the SCI group (Figures 1G1, G2). Data above enhanced the ability of MenSCs in promoting recovery in SCI rats.

### DEGs in SCI group and MenSCs treatment group

**FIGURE 2:**
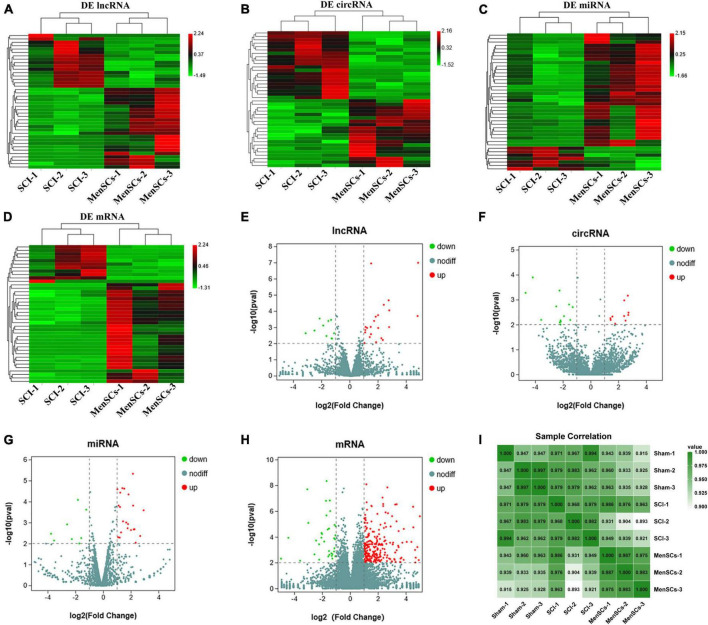
Heatmap and volcano plots of DE ncRNA in SCI and MenSCs treatment groups. **(A–D)** Heatmap of the top 20 up-regulated and down-regulated DE lncRNAs, DE circRNAs, DE miRNAs and mRNAs in the SCI group compared with the MenSCs treatment group. **(E–H)** Volcano plot indicating the differential expression of lncRNAs circRNAs, miRNAs and mRNAs in SCI group and MenSCs treatment group. Up-regulated genes are colored in red, down-regulated genes are colored in Green, respectively. **(I)** All sample Pearson correlation illustrated the correlation between samples among Sham group, SCI group and MenSCs group. The Pearson’s coefficient was calculated on the Transcript per Kilobase per Million mapped reads (TPM) values of RNA species in every combination with Sham, SCI and MenSCs individuals.

To examine the impact of MenSCs treatment in spinal cord injury (SCI) on the expression of non-coding RNAs (ncRNAs) in the epicenter of the lesion, we utilized RNA sequencing (RNA-seq) technology to analyze the total RNA changes in the T10 injured spinal cord. Differential expression genes (DEGs) were identified based on a *P*-value threshold of less than 0.05, yielding a total of 89 upregulated and 65 downregulated long non-coding RNAs (lncRNAs), 65 upregulated and 72 downregulated circular RNAs (circRNAs), 120 upregulated and 74 downregulated microRNAs (miRNAs), and 422 upregulated and 190 downregulated messenger RNAs (mRNAs). To visualize these findings, we constructed heatmaps (Figures 2A–D) and volcano plots (Figures 2E–H) of the top 40 differentially expressed ncRNAs and mRNAs in the SCI and MenSCs treatment groups, and tables were generated to list the top 20 ncRNAs and mRNAs based on their *P*-values (Tables 1–4). Person’s coefficient analysis was used to reveal the relationship of RNA species in every combination using Sham, SCI and MenSCs individual based on TPM values (Figure 2I).

**TABLE 1:** Top 20 differentially expressed lncRNAs in MenSCs treatment tissues comparing with SCI tissues.

Gene id	Status	Log2(FC)	*P*-value	Regulation
MSTRG.11908.3	New	4.857980995	1.03E-07	Up
MSTRG.8083.1	New	1.524141561	1.14E-07	Up
ENSRNOT00000089977	Known	7.167418146	5.52E-07	Up
ENSRNOT00000086791	New	9.770388596	1.04E-05	Up
MSTRG.9869.1	New	2.769961165	2.17E-05	Up
MSTRG.10986.1	New	2.426700417	4.10E-05	Up
MSTRG.9867.1	New	2.809881534	9.04E-05	Up
MSTRG.10694.1	New	2.017572629	0.000179277	Up
ENSRNOT00000086366	New	7.714245518	0.000185048	Up
ENSRNOT00000084080	Known	4.82345823	0.000202159	Up
MSTRG.19848.5	New	−6.906890596	3.55E-08	Down
MSTRG.12339.3	New	−0.948820226	0.000195978	Down
MSTRG.19470.3	New	−0.889612604	0.000220098	Down
MSTRG.9276.1	New	−2.126887618	0.000289522	Down
MSTRG.18724.1	New	−1.315687155	0.000349769	Down
MSTRG.16216.2	New	−1.511252815	0.000412369	Down
MSTRG.11751.3	New	−1.869296308	0.000780809	Down
ENSRNOT00000015084	New	−0.807631911	0.000893813	Down
ENSRNOT00000076019	New	−0.906859113	0.000995149	Down
MSTRG.19848.5	New	−6.906890596	3.55E-08	Down

**TABLE 2:** Top 20 differentially expressed circRNAs in MenSCs treatment tissues comparing with SCI tissues.

Gene id	Source gene	Chrom	Strand	log2(FC)	*P*-value	Regulation
circ_002391	ENSRNOG00000030431	20	+	19.07066934	0.000463713	Up
circ_003705	ENSRNOG00000033693	2	–	18.9617599	0.000673442	Up
circ_002250	ENSRNOG00000000588	20	–	2.680536899	0.000690544	Up
circ_003759	ENSRNOG00000029614	11	+	0.704736774	0.000979995	Up
circ_001055	ENSRNOG00000009345	2	–	2.422588526	0.001064198	Up
circ_009848	ENSRNOG00000023577	9	+	2.728203154	0.003308545	Up
circ_000356	ENSRNOG00000054264	19	+	2.710742505	0.004099427	Up
circ_004090	ENSRNOG00000010248	2	+	18.06686614	0.004293004	Up
circ_008030	ENSRNOG00000004186	6	+	0.621169453	0.00448045	Up
circ_003804	ENSRNOG00000017940	5	+	2.414092902	0.004540641	Up
circ_007672	ENSRNOG00000013422	1	–	−4.17023022	0.000128307	Down
circ_007096	ENSRNOG00000048433	3	+	−0.926647383	0.000131875	Down
circ_003787	ENSRNOG00000025554	8	–	−2.243667929	0.000431083	Down
circ_009862	ENSRNOG00000025160	2	–	−4.692505769	0.00053123	Down
circ_004058	ENSRNOG00000011334	6	+	−18.14523155	0.001104462	Down
circ_006106	ENSRNOG00000053814	10	+	−1.553325707	0.001563391	Down
circ_003397	ENSRNOG00000002919	10	+	−2.47648158	0.001872123	Down
circ_001501	ENSRNOG00000017406	1	+	−1.292439933	0.001989399	Down
circ_000847	ENSRNOG00000010155	16	–	−1.918224766	0.004569152	Down
circ_007218	ENSRNOG00000019754	2	+	−3.562455787	0.006476219	Down

**TABLE 3:** Top 20 differentially expressed miRNAs in MenSCs treatment tissues comparing with SCI tissues.

Gene id	log2(fc)	*P*-value	Regulation
rno-miR-486	2.146972105	4.68E-06	Up
miR-223-y	1.382646319	2.29E-05	Up
rno-miR-223-3p	1.521323407	2.41E-05	Up
rno-miR-146a-3p	1.020186064	2.57E-05	Up
rno-miR-31a-3p	1.230978692	3.70E-05	Up
rno-miR-451-5p	1.829499418	4.53E-05	Up
rno-miR-147	1.114971863	0.00015478	Up
miR-3615-y	2.162853657	0.000154985	Up
rno-miR-146a-5p	0.963968885	0.000168758	Up
rno-miR-155-5p	1.214536423	0.000180881	Up
miR-1983-y	−0.929416449	3.48E-05	Down
rno-miR-184	−1.820941287	8.27E-05	Down
rno-miR-412-5p	−1.219954689	0.000242624	Down
miR-325-x	−5.890527759	0.00029634	Down
rno-miR-144-3p	−0.957597419	0.00058928	Down
novel-m0185-3p	−2.600400881	0.001225686	Down
rno-miR-329-3p	−0.848358845	0.001348461	Down
rno-miR-1298	−0.792763545	0.003190945	Down
novel-m0322-3p	−3.76179312	0.003427587	Down
rno-miR-212-5p	−0.76719471	0.003483157	Down

**TABLE 4:** Top 20 differentially expressed mRNAs in MenSCs treatment tissues comparing with SCI tissues.

Gene id	Symbol	log2(fc)	*P*-value	Regulation
ENSRNOG00000049829	AABR07060872.1	6.612856038	1.96E-18	Up
ENSRNOG00000003666	Jchain	4.93722761	9.73E-16	Up
ENSRNOG00000034190	Ighm	4.881637217	3.86E-11	Up
ENSRNOG00000043451	Spp1	1.16826193	8.29E-09	Up
ENSRNOG00000051690	Clec9a	2.671119144	1.45E-08	Up
ENSRNOG00000046050	Dennd1c	9.437405312	2.14E-08	Up
ENSRNOG00000022009	Mzb1	5.036173613	2.16E-08	Up
ENSRNOG00000022009	Mzb1	5.036173613	2.16E-08	Up
ENSRNOG00000006860	Itk	2.325599077	8.79E-08	Up
ENSRNOG00000006079	Psd4	1.187169948	2.04E-07	Up
ENSRNOG00000058105	Hbb	−1.53037019	9.99E-14	Down
ENSRNOG00000000567	Unc5b	−9.26052755	1.77E-12	Down
ENSRNOG00000019656	Btd	−10.9729797	2.56E-11	Down
ENSRNOG00000009208	Pibf1	−8.92679015	9.27E-11	Down
ENSRNOG00000003065	Glyr1	−1.68627020	4.64E-09	Down
ENSRNOG00000014613	Ddah1	−0.44737263	1.73E-08	Down
ENSRNOG00000019183	Alox15	−3.06726914	2.03E-08	Down
ENSRNOG00000047280	Cttn	−0.54611695	3.43E-08	Down
ENSRNOG00000029886	Hba-a2	−1.45460919	1.49E-07	Down
ENSRNOG00000061299	LOC689064	−1.72381653	1.59E-07	Down

### Verification of ncRNAs and mRNAs expression by qRT-PCR

**FIGURE 3:**
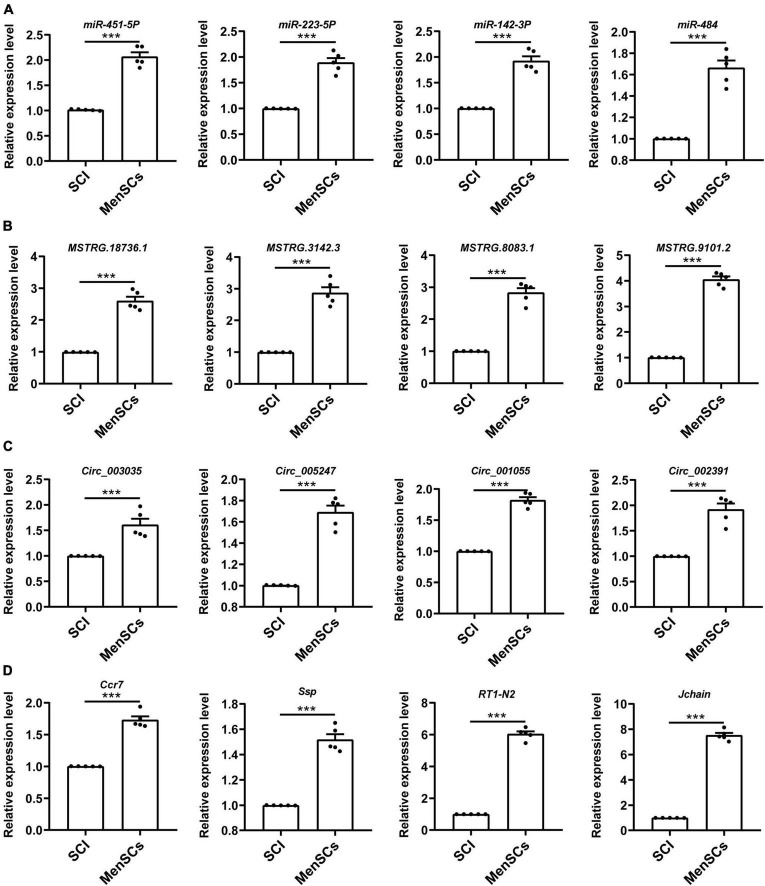
Validation of differential ncRNA and mRNA expression. **(A–D)** Expression of corresponding ncRNAs and mRNAs validated. A total of 16 differentially expressed genes were selected for verification by qRT -PCR analysis, and the results were similar to those obtained from the microarray, *n* = 5. ***P < 0.001.

To validate the reliability of the sequencing data, 12 DE ncRNAs and 4 DE mRNAs were randomly selected to undergo qRT-PCR. These selected ncRNAs and mRNAs from SCI and MenSCs treatment groups included 4 miRNAs (miR-451-5p, miR-223-53p, miR-142-3p, miR-484) (Figure 3A), 4 lncRNAs (MSTRG.18736.1, MSTRG.3142.3, MSTRG.8083.1, MSTRG.9101.2) (Figure 3B), 4 circRNAs (circ_003035, circ_005247, circ_001055, circ_002391) (Figure 3C), and 4 mRNAs (Ccr7, Spp1, RT1-N2, Jchain) (Figure 3D). The expression tendency validated by qRT-PCR was consistent with the results of RNA-seq.

### GO and KEGG pathways enrichment analysis of ncRNA-targeted mRNAs

**FIGURE 4:**
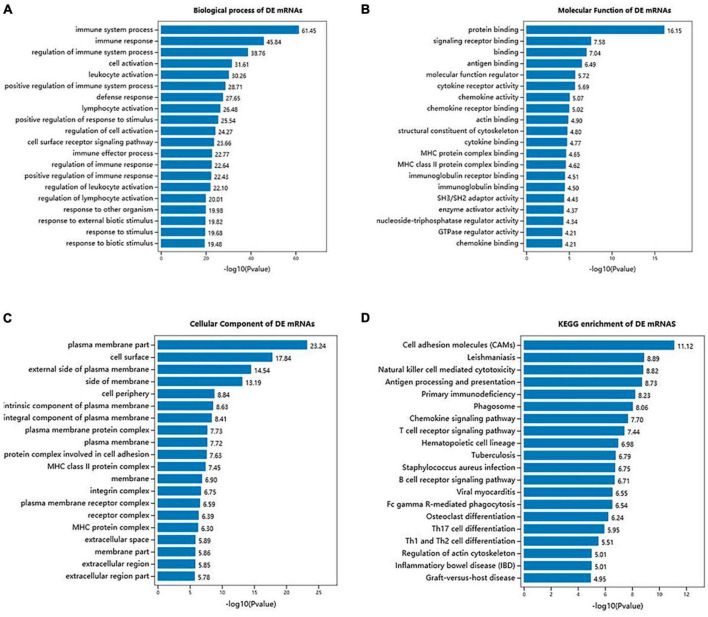
GO terms and KEGG pathways of DE mRNAs in SCI and MenSCs treatment groups. **(A)** Bar graph indicates top 20 significantly enriched GO terms of the DE mRNAs biological process. **(B)** Molecular functions of the top 20 enriched DE mRNAs. **(C)** Cellular component analysis on the top 20 significantly enriched DE mRNAs. **(D)** Exhibition of the top 20 significantly enriched KEGG pathways of DE mRNAs.

To examine the functional enrichment of differentially expressed messenger RNAs (DE mRNAs), we performed Gene Ontology (GO) and Kyoto Encyclopedia of Genes and Genomes (KEGG) pathway analyses using the Omicshare tool. The top 20 terms in each category were selected based on their *P-*values. GO analysis revealed that the biological processes associated with DE mRNAs were primarily related to the immune system process, immune response, regulation of immune system process, cell activation, leukocyte activation, positive regulation of immune system process, defense response, and lymphocyte activation (Figure 4A). Additionally, significant GO terms were observed for the molecular function of DE mRNAs, which included protein binding, signaling receptor binding, binding, antigen binding, molecular function regulator, cytokine receptor activity, chemokine activity, and actin binding (Figure 4B). The cellular components associated with DE mRNAs were mainly related to the plasma membrane part, cell surface, external side of the plasma membrane, side of the membrane, cell periphery, intrinsic component of the plasma membrane, integral component of the plasma membrane, and plasma membrane protein complex (Figure 4C). Moreover, KEGG pathway analysis revealed significant associations between DE mRNAs and various pathways, including cell adhesion molecules (CAMs), leishmaniasis, natural killer cell-mediated cytotoxicity, antigen processing and presentation, primary immunodeficiency, phagosome, chemokine signaling pathway, and T cell receptor signaling pathway (Figure 4D).

### Construction of the ceRNA network

**FIGURE 5:**
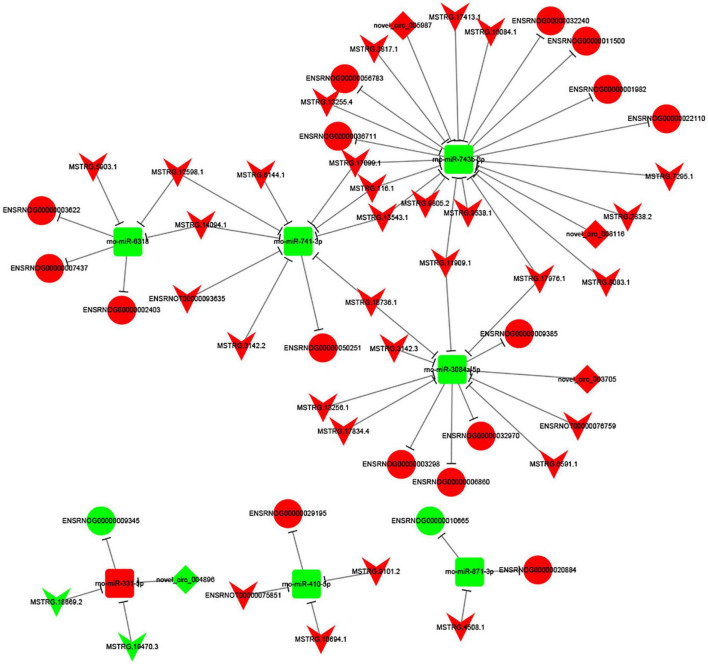
CeRNA network constructed by 44 ncRNAs, 7 miRNAs and 18 target mRNAs. The V node indicates lncRNA, while the square node indicating miRNA, the rhombus node indicating circRNA, and the circular node indicating mRNA. Up-regulated genes are colored in red, down-regulated genes are colored in green, respectively.

To investigate the potential regulatory mechanism underlying the differentially expressed lncRNAs/cirRNAs, miRNAs, and mRNAs, we constructed a complex regulatory network. As depicted in Figure 5, the network was centered around seven miRNAs, which interacted with 44 lncRNAs/circRNAs and 18 target genes. Notably, a single circRNA, circ_004896, was identified to participate in the regulatory network and compete with lncRNA MSTRG.19470.3 and 18869.2 for the target of miR-331-5p. The regulatory network exhibited a high degree of complexity, with some lncRNAs, including MSTRG.11909.1, MSTRG.17976.1, MSTRG.33543.1, MSTRG.17099.1, MSTRG.14094.1, and MSTRG.12598.1, interacting with two miRNAs simultaneously, further complicating the network among the analyzed genes.

### Construction of PPI network and identification of hub genes

**FIGURE 6:**
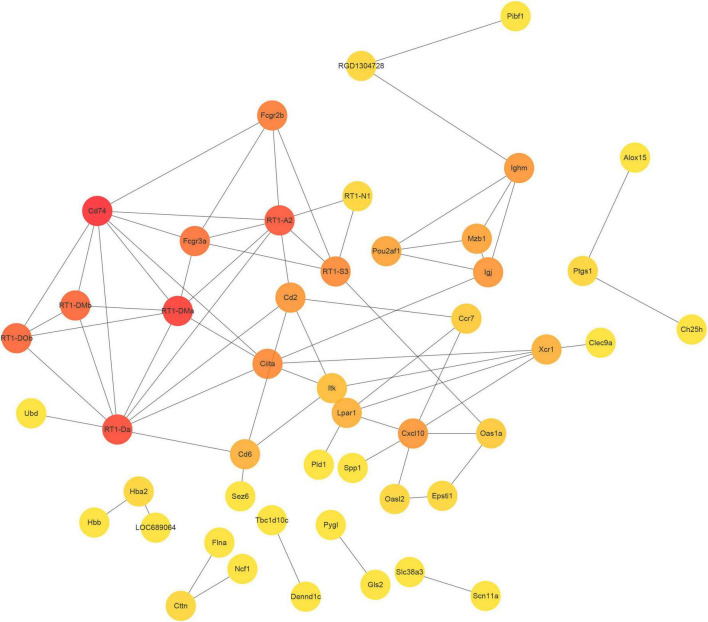
Protein-protein interaction (PPI) network. PPI network constructed by the top 100 DEmRNA-coding protein.

Using the STRING database, we constructed a protein-protein interaction (PPI) network from the top 100 differentially expressed mRNAs, which comprised 47 nodes and 70 edges (Figure 6). To identify the hub genes within this network, we utilized cytoHubba, an extension of Cytoscape software. CD74, RT1-Da, and RT1-A2 were found to be the most highly connected nodes in the network, indicating their potential significance in the biological processes and pathways affected by SCI and MenSCs treatment.

## Discussion

Complex pathogenic processes, including acute and chronic phases, are activated by SCI, resulting in a succession of damaging events, such as inflammation, oxidative stress, ischemia, apoptosis, and motor dysfunction (Anjum et al., 2020). As regenerative medicine has advanced in recent years, stem cell transplantation has become a popular treatment for spinal cord injuries. The transplanted stem cells are capable of secreting nutritional factors, therefore stimulating vascular regeneration, cell differentiation, anti-inflammatory and anti-apoptosis (Gao et al., 2020; Gong et al., 2020; Yamazaki et al., 2020). Nonetheless, the underlying molecular mechanism by stem cell therapy has not yet been well understood.

In this study, we performed RNA-seq to investigate the gene expression pattern in rats with spinal cord injury (SCI) and MenSCs-treated rats 21 days after SCI. The T10 hemisection model was used to induce SCI in the rats, and MenSCs treatment was initiated 7 days after the operation. RNA sequencing was performed on collected specimens. To ensure the reliability and reproducibility of the injury model, we strictly followed a previously established protocol (Wu et al., 2018). Notably, MenSCs treatment resulted in a reduction in the volume of spinal cord lesions, as well as enhanced neuron survival, outgrew axon fibers, decreased CCR7 expression, and improved recovery of motor function. The use of RNA-seq enabled us to comprehensively evaluate the RNAs involved in the injury and treatment processes.

Genome-wide association studies (GWAS) offer a novel perspective on the molecular pathophysiology of diseases (Wang M. H. et al., 2019). Non-coding RNAs (ncRNAs) are enriched in the central nervous system and play a crucial role in growth and development, making them excellent candidates for disease biomarkers and therapeutic targets (Guennewig and Cooper, 2014). ncRNAs are also known to be involved in the key processes of SCI pathophysiology (Ding et al., 2016). In our RNA sequencing analysis, we identified 89 upregulated and 65 downregulated lncRNAs, as well as 65 upregulated and 72 downregulated circRNAs. Additionally, 120 miRNAs were upregulated, and 74 miRNAs were downregulated, while 422 mRNAs were upregulated, and 190 mRNAs were downregulated. These sequencing results were validated by qRT-PCR and were consistent with each other.

To investigate the potential functional implications of the differentially expressed (DE) mRNAs in spinal cord injury (SCI) and MenSCs treatment groups, gene ontology (GO) and Kyoto Encyclopedia of Genes and Genomes (KEGG) enrichment analyses were performed. These analyses were conducted to further elucidate the biological functions of the DE mRNAs. Molecular function analysis revealed that the DE mRNAs were involved in various functions, such as protein binding, signaling receptor binding, antigen binding, molecular function regulator, cytokine receptor activity, chemokine activity, and actin binding. Biological pathway analysis identified cell adhesion and immune system involvement in the process, which supported the construction of cell-cell interaction after injury, promoted axon growth, and reduced growth inhibitors near the injury site (Li R. et al., 2018; Platek et al., 2020). The ceRNA theory was utilized to analyze the regulatory network related to the DE ncRNAs and mRNAs in spinal cord tissue after traumatic injury (Wang N. et al., 2020). Significant changes in the expression of related ncRNAs and mRNAs were observed, and the structure and potential function of the regulatory network were predicted. Among the DE ncRNAs, the eight highest expressed node lncRNAs were selected for further analysis, which included MSTRG.17099.1, MSTRG.116.1, MSTRG.13543.1, MSTRG.11909.1, MSTRG.18736.1, MSTRG.12598.1, MSTRG.14094.1, and MSTRG.17976.1. Furthermore, the target miRNAs of these lncRNAs were predicted through the database. The results showed that MSTRG.17099.1/MSTRG.116.1/MSTRG.13543.1 targeted miR-741-3p, MSTRG.11909.1/MSTRG.18736.1 targeted miR-3084a-5p, and MSTRG.12598.1/MSTRG.14094.1 targeted miR-6318. The RNA sequencing data was verified by quantitative real-time PCR (qRT-PCR) and found to be consistent.

In previous studies, down-regulation of miR-741-3p has been shown to reduce neuronal apoptosis and decrease the expression of pro-inflammatory factors such as IL-6 and TNF-α (Ou et al., 2021). These findings suggest that MSTRG.17099.1/MSTRG.116.1/MSTRG.13543.1-regulated miR-741-3p may have far-reaching significance in SCI, and may represent a potential target for lncRNA-based therapies. By targeting WT1, miR-743a suppresses the proliferation of MM cells *in vitro*, and probably possesses vital functions in kidney development and kidney-associated diseases (Xue et al., 2016). As shown in the gene expression data in the MGI database, miRNA-743 is highly expressed in the head and craniocervical region of mice at embryonic days 12.5–14. However, the expression is diminished afterward. Subsequently, it is highly expressed in the head and craniocervical region of mice from postnatal days 4 to adulthood. Further exploration identified the central nervous system (CNS) of the brain and spinal cord as the main expression sites for miRNA-743, with no expression detected in the peripheral nervous system.^6^ This specific expression pattern, along with its potential involvement in cell proliferation, suggests a potential role in the treatment of spinal cord injury. However, to the best of our knowledge, the potential functions of miRNA-743, miR-3084a-5p and miR-6318 in the context of SCI have not yet been investigated. Further studies are needed to elucidate the potential role of these miRNAs in SCI and their interactions with the identified lncRNAs.

MicroRNAs (miRNAs) play a crucial role in the regulation of gene expression and provide valuable insights into the pathogenesis of various diseases. It has been reported that miRNAs regulate up to 60% of protein-coding genes and participate in various pathological processes (Friedman et al., 2009). For instance, miR-871-3p is involved in regulating cellular signaling and metabolic processes and may affect signaling pathways relevant to insulin secretion and some age-related diseases (Zhang et al., 2020). Similarly, miR-410-5p is involved in the development of diabetic cardiomyopathy and regulates cardiomyocyte apoptosis by targeting PIM1 protein and its downstream protein Bcl-2/Bax (Xia et al., 2020). This miRNA also acts as an endocrine regulator and promotes heart remodeling mediated by metabolic diseases (Zou et al., 2018). Additionally, miR-410-5p is a potential biomarker for prostate cancer and down-regulation of this miRNA can inhibit tumor angiogenesis and tumor growth (Wang et al., 2016, 2017). In the context of stroke, miR-331-5p has been found to mediate the neuroprotective effect of valproic acid-induced middle cerebral artery occlusion in rats after injury (Hunsberger et al., 2012). This miRNA regulates the NLRP3 inflammasome by targeting TRAF6 and may become a novel therapeutic target for ischemic stroke (Zhang H. S. et al., 2019). Furthermore, down-regulation of miR-741-3p can reduce neuronal apoptosis, and therefore, targeting this miRNA may offer a promising strategy for the treatment of SCI.

The analysis of mRNA molecules within the ceRNA network revealed that a majority of the genes had associations with regulating NK cell activity. NK cells serve an “effector” role by releasing cytotoxic granules against xenogeneic cells and an “affecter” role on other immune cells by secreting cytokines. The ceRNA network in current study was enriched with NK inhibitory receptors (KIRs) such as KIR2DL1, KIR2DL3, KIR2DL4, KIR2DS2, and KIR2DS4, as well as NK-activating receptor NKG2D (Liu et al., 2011; Sivori et al., 2019). This gene set is known to be associated with tolerance in xenotransplantation, immunocompetency in endometriosis, reproductive failure, malignant mesothelioma, and acute myeloid leukemia. Additionally, genes such as LILRB1 and Fc alpha receptor (CD89) have also been reported to play a role in immune response as vasculitis (Liu et al., 2022; Woon et al., 2022; Xu et al., 2022). The collection of mRNAs enlisted in the ceRNA network primarily focuses on regulating the inflammatory response function.

In the PPI network, the three hub genes CD74, RT1 class II locus DA (RT1-Da), and RT1-A2 were found. CD74 (Ii) was a type II transmembrane protein that regulated protein transport (Borghese and Clanchy, 2011). Leng et al. (2003) revealed in 2003 that CD74 is a receptor for the cytokine macrophage migration inhibitory factor (MIF). Numerous studies have demonstrated that MIF can regulate the immune system and neural stem cell regeneration via the CD74 pathway (Su et al., 2017; Farr et al., 2020), MIF-CD74 signal can promote the release of chemokine CCL5 from astrocytes, CCL5 promoted the migration of M2-macrophages, and regulated the inflammatory microenvironment in order to maintain homeostasis (Zhang Y. et al., 2019). MIF-CD74 stimulated macrophages by increasing MAPK phosphorylation and increased macrophage phagocytosis to govern regeneration of damaged spinal cord regions (Wang Y. et al., 2019). MIF-CD74 protected against intestine, liver, kidney, and cardiac injury (Farr et al., 2020). However, its involvement in spinal cord damage must be investigated further. RT1-Da contributed to the immune response through antigen processing and presentation of endogenous peptide antigen via MHC class II (Duyar et al., 2005), and was a prototypical MHC class II gene (Ettinger et al., 2004). Under healthy and pathological situations, RT1-A2 regulated the molecular pathways of brain processes such as learning, memory, and synaptic plasticity, according to bioinformatics analyses (VanGuilder Starkey et al., 2012; Fernandes et al., 2018). To confirm the significance of these genes in regulating the positive effects of SCI, however, more experimental verification and modification was required.

Another hub gene, *CCR7*, was identified to be involved in SCI. CCR7, a member of the G protein-coupled receptor family, is found in lymphoid organs and activates B and T cells. It regulates the migration of memory T lymphocytes to inflamed tissues and promotes dendritic cell maturation. CCR7, functioning as a chemokine receptor, significantly influences Tfh cell differentiation, lymphocyte homing, and germinal center formation (Arnold et al., 2007). Markedly upregulated expression of CCR7 was observed in patients suffered with pancreatic cancer, contributing to the enhancement of angiogenesis via chemotactically binding with CCL21 (Zhao et al., 2011). The regulation of Tfh cell differentiation by CCR7 significantly impacts multiple sclerosis relapse (Fan et al., 2015). The expression of CCR7 was found to be decreased in acute SCI patients. In the current study, an upregulation in the transcriptional level and a downregulation in the translational level of CCR7 were observed at day 21 post-surgery. These differences in mRNA and protein levels may be attributed to variances in post-translational modifications. Our research has unveiled the substantial role of CCR7 in the recovery of SCI patients.

In conclusion, based on our research, we have proposed fresh insights that can inspire the creation of novel therapeutic procedures. It should be mentioned, however, that our research is still preliminary, and due to the low number of samples from the sequencing results, additional research and validation are still required.

## Data availability statement

The data presented in the study are deposited in the NCBI SRA repository, accession numbers SRR17599937, SRR17599938, SRR17599939 and NCBI BioProject repository, accession number PRJNA1067278.

## Ethics statement

The animal study was approved by the Institutional Animal Care and Use Committee of Nantong University. The study was conducted in accordance with the local legislation and institutional requirements.

## Author contributions

LQ: Funding acquisition, Investigation, Project administration, Resources, Writing—original draft. WJ: Data curation, Investigation, Methodology, Writing—original draft. WH: Formal analysis, Investigation, Methodology, Software, Writing—original draft. XL: Formal analysis, Methodology, Project administration, Visualization, Writing—original draft. JW: Data curation, Formal analysis, Methodology, Validation, Writing—original draft. SC: Data curation, Formal analysis, Investigation, Methodology, Software, Visualization, Writing—original draft. ZL: Formal analysis, Investigation, Validation, Visualization, Writing—original draft. SY: Formal analysis, Methodology, Software, Validation, Writing—original draft. JL: Methodology, Software, Validation, Writing—original draft. YS: Formal analysis, Methodology, Project administration, Writing—original draft. QFW: Conceptualization, Funding acquisition, Resources, Supervision, Writing—review and editing. CD: Conceptualization, Funding acquisition, Project administration, Supervision, Writing—original draft, Writing—review and editing. QHW: Conceptualization, Funding acquisition, Project administration, Supervision, Writing—original draft, Writing—review and editing.

## Footnotes

1
http://www.bioconductor.org/packages/release/bioc/html/edgeR.html

2
http://www.targetscan.org/

3
http://www.geneontology.org/

4
http://www.genome.jp/kegg/

5
http://string-db.org/

6
https://www.informatics.jax.org/
